# Changes in γδT Cells in Peripheral Blood of Patients with Ulcerative Colitis Exacerbations

**DOI:** 10.1007/s00005-021-00620-x

**Published:** 2021-07-21

**Authors:** Andrzej Gryglewski, Piotr Richter, Marian Szczepanik

**Affiliations:** 1 Department of Anatomy and Department of General Surgery, Gastroenterology, Oncology and Transplantology, Jagiellonian University Medical College, Kraków, Poland; 2 Department of Medical Biology, Jagiellonian University Medical College, Kraków, Poland; 3 Department of General Surgery, Gastroenterology, Oncology and Transplantology, Jagiellonian University Medical College, Kraków, Poland

**Keywords:** Immunosuppression, Ulcerative colitis, Colitis exacerbations, γδT lymphocytes, αβT lymphocytes

## Abstract

The role of γδT cells in ulcerative colitis (UC) is well confirmed in experimental animals and demonstrated in many clinical observations. Recent investigations have indicated that UC is associated with several forms of immune imbalance, such as an imbalance between effector T cells and regulatory T cells. However, little is known about the cellular aspect of clinical colitis exacerbations. We observed 140 patients with histologically confirmed UC over the course of 8 years. We investigated the percentage of γδT and αβT cells in peripheral blood of patients and also the expression of various surface markers (CD25, CD54, CD62L). Patients were assembled into stable colitis and exacerbated colitis groups. The percentage of γδT and αβT cells was evaluated by Ortho Cytorone Absolute flow cytometer. In patients with exacerbated colitis we observed a decrease of γδT cells in peripheral blood and an increased ratio of αβT/γδT. Additionally, we found that exacerbation results in a significant increase of percentage of γδTCD25, γδTCD54 and γδTCD62L lymphocytes in peripheral blood when compared to patients with stable colitis. Exacerbation of ulcerative colitis results in a decreased percentage of γδT cells in peripheral blood with increase of CD25, CD54 and CD62L expressing γδT cells. This may represent the effect of cell activation and migration, similar to that observed after the surgical trauma. We hope that this observation may help to predict exacerbations in colitis patients.

## Introduction

Inflammatory bowel disease (IBD) in humans, such as ulcerative colitis (UC) and Crohn’s disease (CD), are chronic, relapsing inflammatory diseases of the digestive tract. UC is a chronic inflammatory disease of the colon with largely unknown causes and affects approximately 900,000 people in the United States, 1.5 million people in Europe, and over three million people worldwide, mainly of Caucasian descent (Armuzzi et al. 2020). UC onset occurs predominantly in young adults aged 20–40 years, with no difference between males and females (Ji et al. 2016).

The disease when diagnosed can be classified as stagnant or exacerbated. Occurring UC exacerbations deteriorate patients’ life quality and increase the risk of complications. The pathogenesis of UC involves genetic factors, an aberrant immune reaction, environmental factors, and intestinal microbiota (Hanauer 2006; Sartor 2006). The immune response reflects defects in both innate and adaptive immunity. Defects of the innate immune response lead to inappropriate responses to commensal gut microbiota including the production of various cytokines promoting Th2-mediated immune response (Mayer 2010; Sanchez-Munoz et al. 2008). More recent findings show enhanced expression of thymic stromal lymphopoietin in mucosal lesions of UC patients in which Th2 cytokine production is predominant (Tanaka et al. 2010). Interestingly, Pastorelli et al. (2010) showed a specific increase of mucosal interleukin (IL)-33 in active UC suggesting that this cytokine may be a critical mediator in the pathogenesis of UC. Presently, we provide the better quality of life of patients, by preventing disease recurrence using a variety of medications. This increases the importance of early diagnosis of disease recurrence.

There is a large number of animal experiments that hint at the etiology of UC. These studies describe the pathological behavior of various immune cells in animal models of UC. Interestingly, intrarectal trinitrobenzene sulfonic acid administration to SJL/J or C57BL/10 mice induces a transmural colitis mainly driven by a Th1-mediated immune response and characterized by infiltration of the lamina propria with CD4^+^ T cells, neutrophils, and macrophages, whereas oxazolone colitis is characterized by robust IL-13 production originating from lamina propria CD4^+^ natural killer T cells (Kiesler et al. 2015). A major step forward in the development of murine models of intestinal inflammation came with the discovery that adoptive transfer of naïve CD4^+^ T cells (CD4^+^CD45RB^high^ T cells) from donor mice into syngeneic immunodeficient SCID or Rag1^−/−^ recipient mice causes a wasting disease and a primarily colonic inflammation (Kiesler et al. 2015). Severe colitis could, however, be induced in nude mice by transfer of activated/Th1 CD4^+^CD45RB^low^ T cells, whereas intraepithelial lymphocytes may play an important role in suppressing the development of chronic colitis in this model (Laroux et al. 2004). Nevertheless, transcription factor T-box (T-bet) deficient Th cells efficiently induce colitis, as reflected by weight loss, diarrhea, and colon histopatology. Interestingly, T-bet deficient Th cells differenciate into Th1/Th17 cells, able to express interferon-γ and IL-17A upon restimulation (Zimmermann et al. 2016). Most authors agree that γδT cells play an important role in inflammatory response in the colon (Johnson et al. 2020; McCarthy and Eberl 2018). T lymphocytes can be divided into two sub-populations according to the antigen receptor they express: the αβT cells and γδT cells. αβT cells involved in adaptive immune response are the largest population of T lymphocytes in peripheral blood, where they account for up to 95% of circulating T cells (Catalan-Serra et al. 2017). In both humans and mice, γδT cells comprise a minor part (1–5%) of the circulating T cell compartment found in blood and secondary lymphoid organs (Nielsen et al. 2017). These cells belong to the first line of defense in the epithelia, as well as they play an important role in immunoregulation. γδT cells can be classified into three populations: Vδ1^+^, Vδ2^+^ (also known as Vγ9 Vδ2) and Vδ3^+^. Vδ1^+^ T cells are predominant in epithelia and play a crucial role in epithelial regeneration, whereas Vδ2^+^ T cell population is predominant subtype in peripheral blood and has cytotoxic activity (Catalan-Serra et al. 2017). Vδ3^+^ T lymphocytes are present in the intestinal mucosa and they constitute only about 0.2% of T lymphocytes in the peripheral blood (Catalan-Serra et al. 2017). It was shown that blood from CD patients included an increased percentage of gut-tropic integrin β7-expressing Vδ2^+^ T cells, while “Th-committed” CD27-expressing Vδ2^+^ T cells were selectively depleted. A corresponding population of CD27^+^Vδ2^+^ T cells was present in mucosal biopsies from CD patients and produced elevated levels of tumor necrosis factor (TNF)-α compared with controls (McCarthy et al. 2015). γδT lymphocytes can attack target cells directly through their cytotoxic activity or indirectly through the activation of other immune cells. γδT lymphocytes respond upon the recognition of stress antigens, which promotes cytokine production and regulates pathogen clearance, alleviates inflammation, and promotes tissue homeostasis in response to stress. Tissue-resident epithelial γδT lymphocytes represent a major T cell population in epithelia and are ideally positioned to perform barrier surveillance and aid in tissue homeostasis and repair (Lee and Baldridge 2017). T cell receptor (TCR)γδ-bearing murine dendritic epidermal T cells are involved in the regulation of epidermal integrity and promote wound healing in the skin (Jameson et al. 2002). Intestinal intraepithelial γδT cells regulate intestinal epithelial homeostasis (Boismenu and Havran 1994; Komano et al. 1995). γδT cells are also important in immune surveillance of the epithelium by providing the first line of defense against infectious pathogens attacking body surfaces and in regulating innate and adoptive immunity (Hayday and Tigelaar 2003; Komano et al. 1995; Mak and Ferrick 1998; Shiohara et al. 1996). Furthermore, γδT cells appear to down-regulate robust αβT cell-driven immune responses that often result in severe immunopathology (McVay et al. 1997). γδT cells in the diseased mucosa have also been documented in UC patients (Yeung et al. 2000).

Recent investigations showed association of UC with an imbalance between effector T cells and regulatory T (Treg) cells. Interestingly, some clinical observations suggest the contribution of γδT cells to the development of UC (Johnson et al. 2020; Mayer 2010; McCarthy and Eberl 2018).

Very little is known about colitis exacerbations especially from a clinical perspective. Exacerbations cannot be predicted based on preclinical animal models. However, it has been already reported that γδT cells are involved in the pathogenesis of CD. Kadivar et al. (2016) showed that γδT cells expressing both CD8α and CD8β constitute a novel lymphocyte subset, which is strongly enriched within the gut and may play an important role in gut homeostasis and mucosal healing in IBD. Interestingly, Andreu-Ballester et al. (2011) found a decrease of lymphocytes in the peripheral blood of patients with CD. This decrease was more evident and significant in the γδT lymphocyte subsets, both overall and independently of the subsets of patients studied. CD8^+^ T lymphocytes were lowest in both the γδT and αβT subsets (Andreu-Ballester et al. 2011). From an immunological point of view, the cause of exacerbation might be an increase in the pathological processes associated with the course of colitis, or a separate, as yet unknown pathological triggering process (Hanai et al. 2013).

Our previous experiments in mice demonstrated that surgical trauma in the abdomen induces γδT lymphocyte migration from peripheral blood to peritoneal lymphoid organs (Gryglewski and Szczepanik 2017). Similarly we found in human that major surgery located in the peritoneal cavity (gastric and colorectal surgery) decreases the percentage of γδT cells in peripheral blood. We suspect that similar process of T cell migration may occur during UC exacerbations (Gryglewski et al. 1997; Gryglewski and Szczepanik 2017).

To verify our hypothesis, we analyzed the difference between αβT and γδT cell numbers in the peripheral blood of patients with disease in remission or flaring, compared to healthy controls.

## Materials and Methods

### Obtaining Blood Samples

Five ml of venous blood was obtained from each patient. Each sample was immediately processed and analyzed.

### Reagents

The following monoclonal antibodies (mAbs) were used: anti-TCRα/β-FITC (clone WT 31), anti-TCRγ/δ-1-FITC (clone 11F2), anti-CD25-PE (clone M-A251), anti-CD54-PE (clone LB-2) and anti-CD62L-PE (clone DREG-56). All mAbs and 10X concentrate FACS Lysing Solution were purchased from BD Biosciences (San Jose, CA, USA).

### Flow Cytometric Staining and Cell Analysis

Fifty μl of whole blood was mixed with 20 μl of the appropriate fluorochrome-conjugated mAb and incubated for 30 min at room temperature. The following staining panels were used: TCRαβ-FITC, TCRγδ-FITC, TCRγδ-FITC/CD25-PE, TCRγδ-FITC/CD54-PE and TCRγδ-FITC/CD62L-PE. The percentage of presented cell populations is referred to total analyzed lymphocytes.

When staining was finished, 450 μl of 1× FACS Lysing Solution was added and samples were incubated for 20 min in the dark at room temperature. Then samples were washed and resuspended in PBS containing 1% paraformaldehyde. All flow cytometric analyses were conducted within 2 h of fixation. Samples were run on Ortho Cytorone Absolute flow cytometer (Ortho Diagnostic Systems, Raritan, NJ). Immunocount II software was used for data acquisition and analysis.

### Statistical Analysis

Data were analyzed using Statistica 10.0 PL software (licensed to Jagiellonian University Medical College). Descriptive statistics were used (mean, standard deviation, percentage distribution). Statistical significance was determined by *t* test (*p* < 0.05).

### Patients’ Characteristics

One hundred and forty patients with histologically confirmed UC were included in this prospective case control study from 1999 to 2007. We recruited UC patients admitted to the outpatient gastroenterology and surgery clinic of I Department of General Surgery, Gastroenterology, Oncology and Transplantology, exhibiting abdominal pain and bloody or mucous diarrhea. The majority of the patients studied were newly diagnosed, and some already treated with sulfosalazopirin and/or acetylsalicylic acid. We excluded individuals with prior cytostatic or steroid treatment from the study to avoid bias due to the effect of these drugs on peripheral blood lymphocyte counts. All patients underwent colonoscopy with UC confirmed histologically. At that time patients were divided into stable and exacerbated groups. Disease activity was measured using the Truelove, Witts and Montreal scale. For exacerbations of the disease, we monitored for severe symptoms according to the scale (Satsangi et al. 2006; Truelove and Witts 1955). Exclusion criteria included no consent from the patient or a history of steroid or cytostatic medication. Peripheral blood samples for immunological tests were obtained on the first day after accepting the patient for observation. All patients had a regular follow up and were instructed to contact the clinic every three months or if they experienced any complications (data not presented). We also recruited an additional 21 patients who developed UC exacerbations to the study over a three-year period. Blood samples were taken for immunological tests from these participants and their results were added to the “exacerbation group”, unless they were already treated with steroids or cytostatic drugs e.g. azathioprine. All other patients were included within the stable colitis group. Laboratory staff members (7) and patients with uncomplicated gallstones (50) prepared for surgery participated as healthy controls (Table 1).

**Table 1:** Patients’ characteristics

Patients’ group	Number	Gender M/F	Age (years)	Disease duration (months)	Montreal classification (extent) E1/E2/E3	ASA/SSZ (ever)
Stable colitis	88	41/47	36 ± 12	63 ± 37	30/25/33	57
Exacerbations	52	23/29	43 ± 15	46 ± 32	9/23/20	38
Control	57	30/27	51 ± 17			
Total	197	94/103	43.3 ± 14.6			

*E* Extent according to the Montreal classification (E1—ulcerative proctitis, E2—left sided UC (distal UC), E3—extensive UC (pancolitis)), *ASA/SSZ* (ever) acetylsalicylic acid/sulfosalazopirin ever prescribed

### Ethical Considerations

The research protocol was approved by the Jagiellonian University Ethics Committee (registry number KBET/130/B/2014). The study was in accordance with the ethical standards defined in the1964 Declaration of Helsinki and its amendments.

## Results

Flow cytometry was used to determine the differences of αβT and γδT lymphocytes in peripheral blood between different groups of colitis and the healthy controls.

**Fig. 1:**
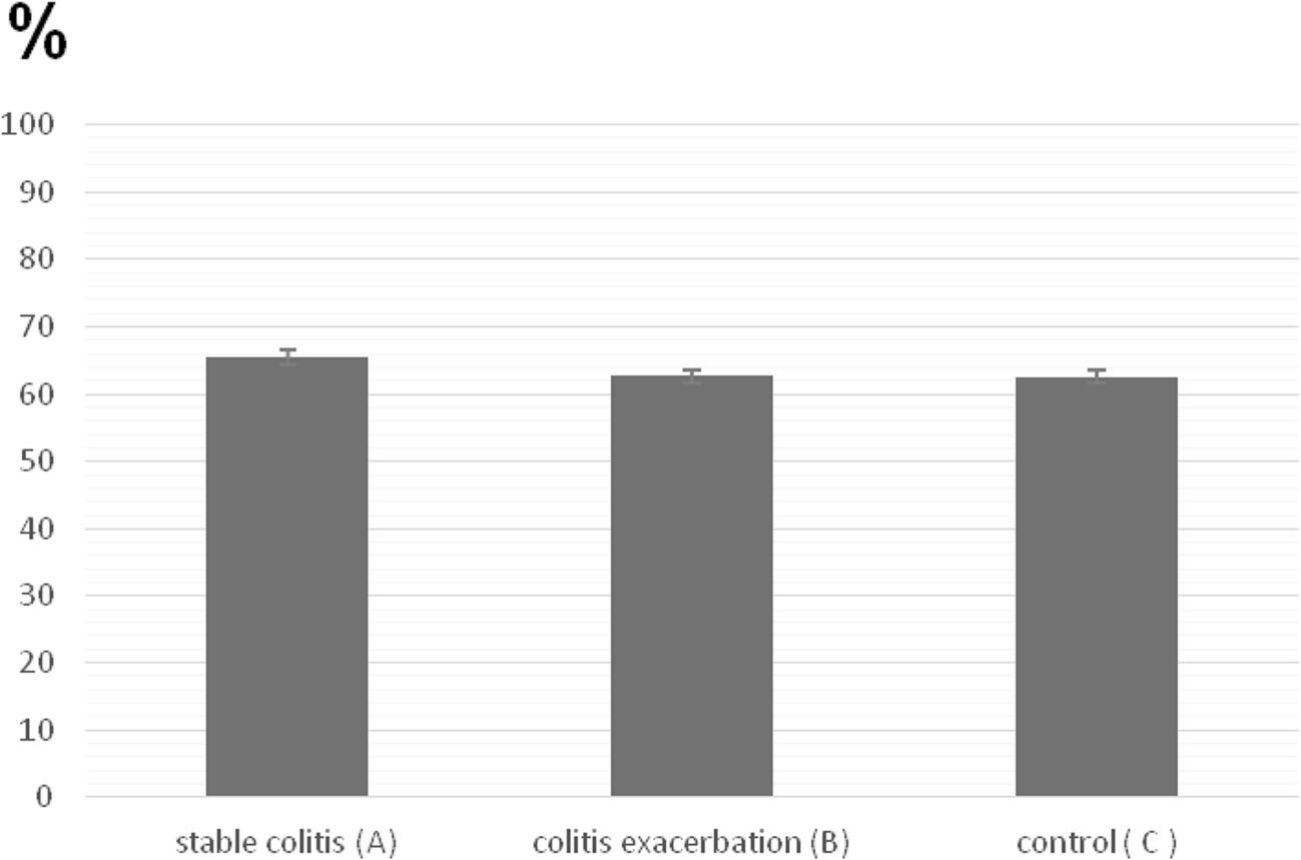
The percent of αβT cells in peripheral blood. Blood samples from patients with stable colitis (**A**), colitis exacerbation (**B**) and from control group (**C**) were stained with anti-TCRα/β-FITC mAb prior to FACS analysis. Group A: *n* = 88, Group B: *n* = 52, Group C: *n* = 57. All error bars represent SD. *p* = NS (Student’s *t* test)

Data presented in Fig. 1 and Table 2 show that colitis exacerbation does not affect the percentage of αβT cells (62.7 ± 1.6; Group B) when compared to the percentage of αβT cells in patients with stable colitis (65.5 ± 1.4; Group A) and in controls (62.6 ± 2.4; Group C), (Group B vs. A and C; *p* = NS).

**Table 2:** The percentage of αβT and γδT cells in peripheral blood from patients with stable colitis (A), colitis exacerbations (B) and control (C)

	The percentage of αβT cells	The percentage of γδT cells	The ratio of αβT/γδT cells
Stable colitis	65.5 ± 1.4	7.3 ± 0.3	9.0 ± 3.0
Colitis exacerbations	62.7 ± 1.6	2.0 ± 0.1	31.35 ± 3.2
Control	62.6 ± 2.4	5.2 ± 0.25	12.0 ± 1.6
Statistical significance	–	B vs. A and C *p* < 0.001	B vs. A and C *p* < 0.001

**Fig. 2:**
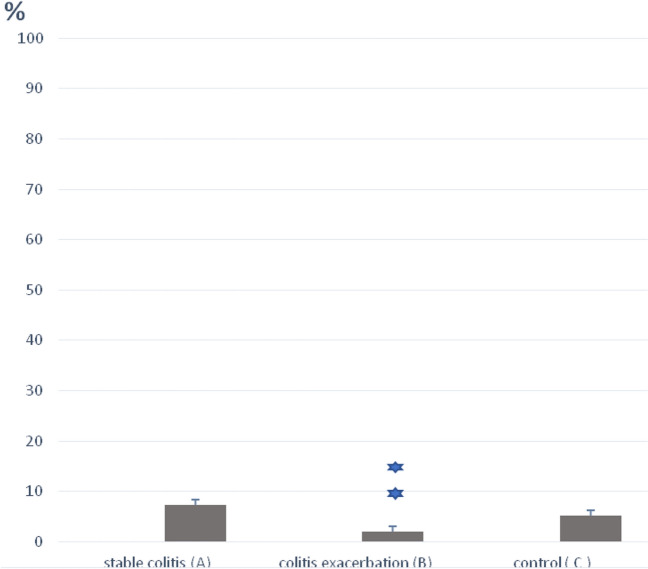
The percent of γδT cells in peripheral blood. Blood samples from patients with stable colitis (**A**), colitis exacerbation (**B**) and from control group (**C**) were stained with anti-TCRγ/δ-1-FITC mAb prior to FACS analysis. Group A: *n* = 88, Group B: *n* = 52, Group C: *n* = 57. All error bars represent SD. **p < 0.001 (Student’s *t* test)

Interestingly, results presented in Fig. 2 and Table 2 show differences between the γδT in the exacerbations of colitis, stable colitis and control group. They show that the percentage of γδ Tcells was significantly lower in the peripheral blood of patients with colitis exacerbations (2.0 ± 0.1; Group B) compared to the control group (5.2 ± 0.25; Group C) and the patients with stable colitis (7.3 ± 0.3; Group A), (Group B vs. A and C, *p* < 0.001).

**Fig. 3:**
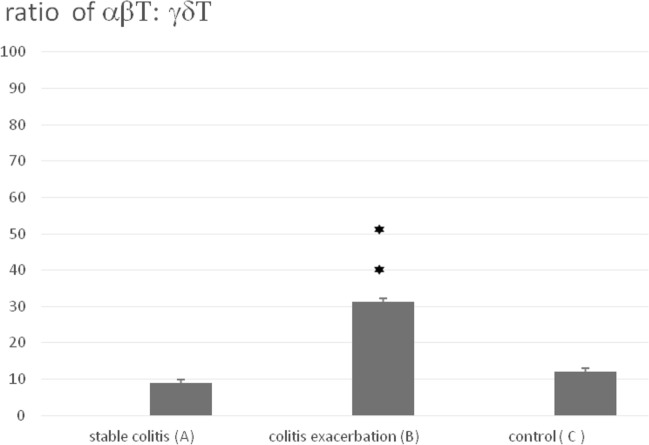
The ratio of αβT to γδT cells in peripheral blood. Blood samples from patients with stable colitis (**A**), colitis exacerbation (**B**) and from control group (**C**) were stained with either anti-TCRα/β-FITC or anti-TCRγ/δ-1-FITC mAb prior to FACS analysis. Group A: *n* = 88, Group B: *n* = 52, Group C: *n* = 57. Then, the ratio of αβT to γδT cells was evaluated. All error bars represent SD. **p < 0.001 (Student’s *t* test)

Additionally, in Fig. 3 and Table 2 we compared the ratio of αβT/γδT lymphocytes in peripheral blood from patients with stable disease, diagnosed colitis exacerbations, and in the controls. The ratio αβT/γδTwas significantly higher in the group of patients with colitis exacerbations (31.35 ± 3.2; Group B) than the ratio αβT/γδT in the group of patients with stable colitis (9.0 ± 3.0; Group A), and the controls (12.0 ± 1.6; Group C), (Group B vs. A and C, *p* < 0.001).

In addition, we evaluated cumulative counts of various cell surface markers on γδT cells in the peripheral blood of patients with stable or exacerbated colitis and in controls.

**Fig. 4:**
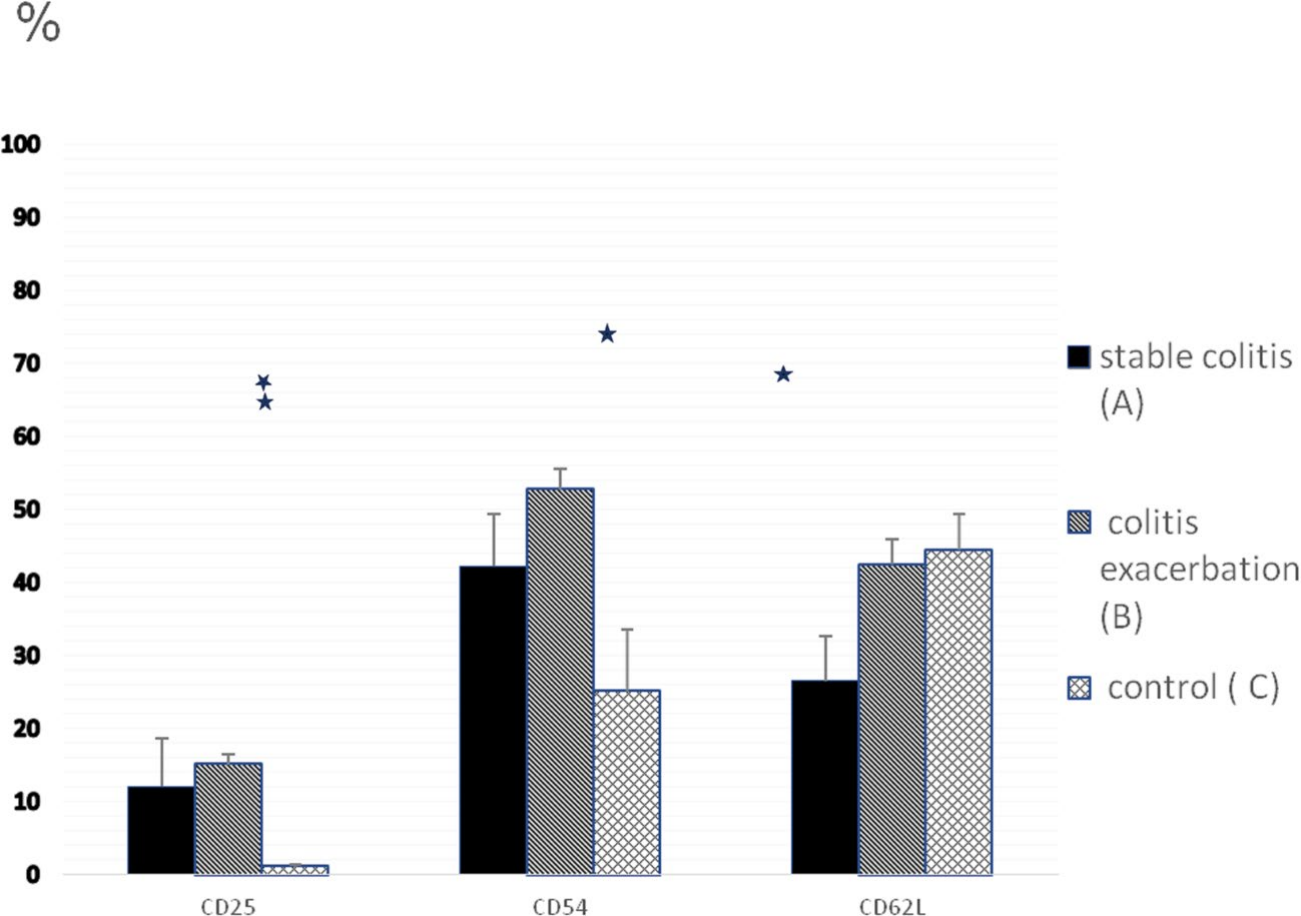
The percentage of γδT lymphocytes expressing various surface markers (CD25, CD54, CD62L) in peripheral blood. Blood samples from patients with stable colitis (**A**), colitis exacerbation (**B**) and from control group (**C**) were stained with anti-TCRγ/δ-1-FITC and anti-CD25-PE or anti-CD54-PE or anti-CD62L-PE mAb prior to FACS analysis. Group A: *n* = 88, Group B: *n* = 52, Group C: *n* = 57. All error bars represent SD. *p < 0.01, **p < 0.001 (Student’s *t* test)

To characterize T cell activation profile of γδT lymphocytes, cells were additionally stained for expression of the following cell surface markers: CD25, CD54 and CD62L. The data presented in Fig. 4 and Table 3 show the cumulative counts of γδT lymphocytes expressing various surface markers in peripheral blood from patients with colitis exacerbations compared to the stable colitis group and the control group. We found increased percentage of γδT CD25 both in stable (12.06 ± 6.6; Group A) and exacerbated colitis (15.08 ± 1.3; Group B) groups when compared to the control group (1.1 ± 0.3; Group C), (Groups A and B vs. C, *p* < 0.001).

**Table 3:** The percentage of γδT lymphocytes expressing various surface markers in peripheral blood from patients with colitis stable colitis (A), colitis exacerbations (B) and control (C)

	Percentage of γδT CD25 cells	Percentage of γδT CD54 cells	Percentage of γδT CD62L cells
Stable colitis	12.06 ± 6.6	42.2 ± 7.1	26.5 ± 6.2
Colitis exacerbation	15.08 ± 1.3	52.7 ± 2.8	42.4 ± 3.6
Control	1.1 ± 0.3	25.1 ± 8.4	44.4 ± 5.0
Statistical significance	A and B vs. C *p* < 0.001	A and B vs. C *p* < 0.01	B and C vs. A *p* < 0.01

Similarly percentage of γδT CD54 cells was significantly increased in stable (42.2 ± 7.1; Group A) and exacerbated (52.7 ± 2.8; Group B) colitis groups compared to the control group (25.1 ± 8.4; Group C), (Groups A and B vs. C, *p* < 0.01).

We observed lower percentages of γδT cells expressing CD62L in the group of patients with stable colitis (26.5 ± 6.2; Group A) compared to colitis exacerbation (42.4 ± 3.6; Group B) and the control group (44.4 ± 5.0; Group C), (Group A vs. B and C; *p* < 0.01).

## Discussion

There is a growing evidence supporting the idea that γδT cells play an active multifaceted immunoregulatory role in the coordinated innate and adoptive immune responses to maintain the integrity of many organs containing epithelia especially in colon tissue (Boismenu and Havran 1994; Hayday and Tigelaar 2003; Jameson et al. 2002; Komano et al. 1995; Mayer 2010; Yeung et al. 2000). Recent investigations have indicated that UC is associated with several forms of immune imbalance, such as an imbalance between effector T cells and Treg cells (Havran 2000; Tonegawa et al. 1989).

In this study we found significant decrease of γδT but not αβT cells in the peripheral blood of patients with colitis during the exacerbation of the disease compared to the stable disease and the control group. In many publications the increased γδT cell number has been documented in diseased mucosa probes in UC patients (Gryglewski et al. 1997; Hanai et al. 2008; Koch et al. 2010; Yu et al. 2007). Thus it is possible that observed decrease of γδT cells in the peripheral blood in patients with colitis exacerbation results from the relocation of these cells to the diseased gut mucosa. Our observation of cells involved in immunoregulation, circulating from peripheral blood to mucosal tissue, and to other immunologically active organs is confirmed in the literature. Eastaff-Leung et al. (2010) report a decreased number of peripheral blood Treg cells associated with a reduced ratio of Treg to Th17 cells in peripheral blood. Interestingly, increased intestinal Foxp3 expression was observed in IBD patients what may suggest relocation of Treg cells from peripheral blood to the intestines (Eastaff-Leung et al. 2010). Based on our present observations it is difficult to define precisely the behavior of γδT cells during the exacerbation of colitis, mainly because the blood sample was collected once at unknown phase of exacerbation. Our observation allows only for general evaluation of the level of γδT and αβT cells and provides the general difference between their level in exacerbations and stable colitis or remissions. It is important to note that some patients were treated pharmacologically and some of drugs (especially steroids and cytostatic agents) may influenced different blood cell levels. To avoid this, we excluded patients treated with steroids or cytostatics or with biologic therapy. Despite of this the fact that most of our patients were treated with the sulphasalazine or mesalazine may influence final results, and should be taken into the consideration. Nevertheless, our observation of patients with the exacerbation shown decreased γδT blood cell numbers comparing to patients with stable colitis or controls, strongly suggesting these cells participation. There are reports showing involvement of γδT cells in course of IBD. Interestingly, the deficit of γδT cells was related with the severity and the risk for surgical relapse in CD patients (Andreu-Ballester et al. 2020). Additionally, animal studies show that resident γδT cells are an early, innate‐like source of IL‐17 and that these cells amplify Th17 responses and exacerbate colitis development (Yurchenko et al. 2011). Moreover, it was observed that γδT cells directly promote the generation of gut antigen-reactive T effector cells (Do et al. 2014). Thus, it is possible that observed in our study reduction of γδT cells in peripheral blood during disease exacerbation might be caused by γδT lymphocyte migration from peripheral blood to peritoneal lymphoid organs and subsequent participation in disease progression. This is in line with our previous animal (Gryglewski et al. 1997) and human studies showing that surgery located in the peritoneal cavity (gastric and colorectal surgery) decreases the percentage of γδT cells in peripheral blood (Gryglewski and Szczepanik 2017). Interestingly, in the group that had a large decrease in γδT cells we found significantly more septic complications than in the group of patients with small γδT cell decrease (Gryglewski et al. 2020). We would like to underline that in our work we evaluated percentage of all γδT cells without describing existing populations. As we mentioned in Introduction, γδT cells can be classified into three populations: Vδ1^+^, Vδ2^+^ and Vδ3^+^(Catalan-Serra et al. 2017; Davey et al. 2017). Interestingly, there are reports showing that human Vδ2^+^ T lymphocytes comprise two separate subsets, which have different biological activity. Vγ9^+^ Vδ2^+^ T lymphocytes appear to represent an innate-like subset which is phosphoantigen-reactive, whereas Vγ9^–^ Vδ2^+^ population represents unconventional T cell compartment that exhibits many of the hallmarks of an adaptive immunity (Davey et al. 2018). Further work is required to determine if γδT cells investigated in our work exhibit hallmarks of innate-like or adaptive immunity. In addition, our analysis of various surface markers on γδT cells shows increased percentage of γδT CD25-positive cells in stable UC, as compared to controls, and an even greater increase of γδT CD25-positive cells in colitis exacerbations. CD25 is the alpha chain of the IL-2 receptor which is a marker of the state activation of both αβT and γδT cell subsets induced by interactions of the TCR with its ligands (Migalovich Sheikhet et al. 2018). It was shown that IL-2 plays a role in generation of γδT cells (Malik et al. 2016). Thus, increased expression of CD25 in both stable colitis and colitis exacerbation groups compared to control group may suggest increased activity of γδT cells in UC patients despite of disease status.

Comparing expression of CD54 cell marker on γδT-positive cells, we found relatively higher percentage of CD54 expressing γδT cells in colitis remission and even higher in exacerbations, when compared to the controls. CD54 (ICAM-1) plays an important role in leukocyte trafficking, immunological synapse formation, and numerous cellular immune responses. It is suggested that CD54 on T cells does not participate in T cell migration, but rather signals delivered into the T cell through CD54 are more likely involved in cell recognition or activation. Indeed, Chirathaworn et al. (2002) found that signaling of resting T cells through CD54 delivers a distinct costimulatory signal resulting in T cell activation and proliferation. Thus increased expression of CD25 and CD54 on γδT cells in colitis patients may suggest activation of these cells and their involvement in the disease. Finally, we found significant decrease of CD62L expressing γδT cells in blood samples from patients with colitis remission when compared to patients with colitis exacerbation and the control group. CD62L expression on naive T cells is required for their efficient recirculation and compartmentalization between blood and lymph nodes (Hengel et al. 2003). Interestingly mucosal healing was reflected by significant increase of CD62L expression in mucosal T cells in patients in remission compared to those with active inflammation (Karlsson et al. 2014). Thus significant decrease of CD62L expressing γδT in blood samples from patients with colitis remission may be resulting from the relocation of these cells to the gut mucosa. More recently, several other candidate cell-based biomarkers have emerged: CD8CD69 in both IBDs, CD4CD45RO and CD4HLADR in CD and CD4, Th2, Th17 and CD8HLADR in UC seem to be promising markers of remission or of the prediction of a therapeutic response to anti-TNF treatment (Dulic et al. 2020). Interestingly, Andreu-Ballester et al. (2020) found a relationship between lower γδT cell levels and risk of surgical relapse in CD. The lowest subsets observed in CD patients with surgical relapse were CD3^+^γδ, CD3^+^CD8^+^γδ and CD3^+^CD56^+^γδ T cells, whereas the lowest levels of CD3^+^γδ and CD3^+^CD8^+^γδ T lymphocytes were observed in the fistulizing phenotype (Andreu-Ballester et al. 2020).

In summary, our observations indicate that the change in the number of γδT lymphocytes in the peripheral blood occurs in patients during an exacerbation of UC. Understanding the temporal relationship between γδT cells and the exacerbation of colitis may identify important biomarkers for earlier diagnosis of disease flares. The more permanent observation of γδT cells in peripheral blood of patients with colitis might help in early recognition and treatment of exacerbations in patients with UC.

## Conclusion

Exacerbation of ulcerative colitis results in a decreased percentage of γδT cells in peripheral blood with increase of CD25, CD54 and CD62L expressing γδT cells. This may represent the effect of cell activation and migration, similar to that observed after the surgical trauma. We hope that this observation may help to predict exacerbations in colitis patients.

## Abbreviations

UC: Ulcerative colitis
CD: Crohn’s disease
IBD: Inflammatory bowel disease
TCR: T cell receptor
mAbs: Monoclonal antibodies
